# Glycosylation of uroplakins. Implications for bladder physiopathology

**DOI:** 10.1007/s10719-014-9564-4

**Published:** 2014-11-15

**Authors:** Iwona Kątnik-Prastowska, Jolanta Lis, Agata Matejuk

**Affiliations:** Department of Chemistry and Immunochemistry, Medical University of Wroclaw, Bujwida 44a, 50-345 Wroclaw, Poland

**Keywords:** Uroplakins, Urothelium, Glycosylation, Glycomarkers, Urothelial pathologies

## Abstract

Urothelium, a specialized epithelium, covers the urinary tract and act not only as a barrier separating its light from the surrounding tissues, but fulfills an important role in maintaining the homeostasis of the urothelial tract and well-being of the whole organism. Proper function of urothelium is dependent on the precise assemble of highly specialized glycoproteins called uroplakins, the end products and differentiation markers of the urothelial cells. Glycosylation changes in uroplakins correlate with and might reflect progressive stages of pathological conditions of the urothelium such as cancer, urinary tract infections, interstitial cystitis and others. In this review we focus on sugar components of uroplakins, their emerging role in urothelial biology and disease implications. The advances in our understanding of uroplakins changes in glycan moieties composition, structure, assembly and expression of their glycovariants could potentially lead to the development of targeted therapies and discoveries of novel urine and plasma markers for the benefit of patients with urinary tract diseases.

## Introduction

**Fig. 1:**
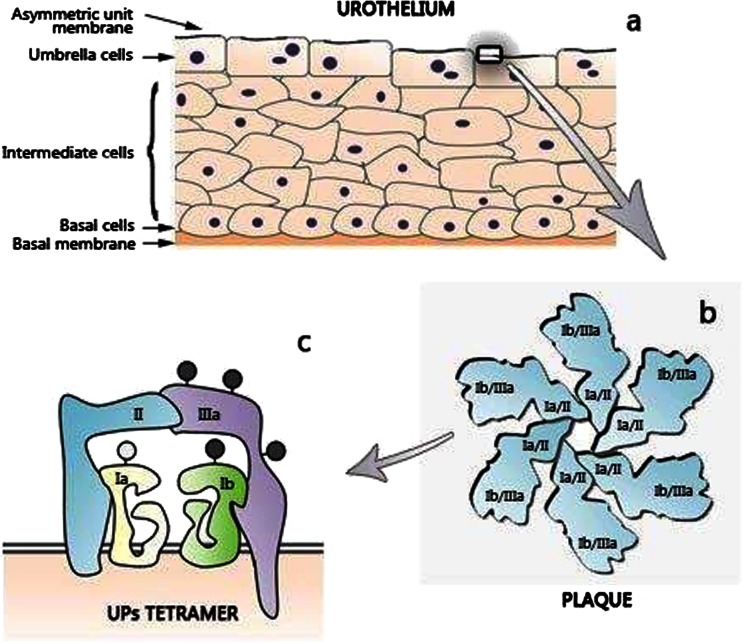
Scheme of the urothelium and uroplakin plaque composition. Urothelium is composed of three layers: a basal cell layer attached to a basement membrane, an intermediate layer, and an apical layer with large hexagonal cells known as umbrella cells (**a**). The apical surface of umbrella cells creates a unique asymmetric unit membrane (AUM) composed of hexagonal plaques with six tetramers where two dimers UPIa/II and Ib/IIIa are linked (**b**). High-mannose N-glycan (*light gray circle*) on UPIa and complex N-glycans (*dark gray circle*) on UPIb and IIIa are depicted. Mature UPII lacks sugar moieties (**c**). Drown based on [22, 46]

Urothelium is a multilayer epithelium of the inner surface of the mammalian urinary bladder which extends from the renal pelvis to the urethra. It is composed of three to six layers of heterogeneous epithelia cells at different stages of differentiation, that contain basal, intermediate and umbrella cells (Fig. 1a) [1–3]. Urothelium is an unusual epithelial tissue because of stratified complex of cellular layers with intimate connections to neural and connective tissue elements [4, 5]. The mammalian urothelium apical surface is in 90 % covered by urothelial plaques [6] forming the membrane interdigitation at the cell borders [7]. This interdigitation creates a membrane zipper, likely contributing to the barrier function of the urothelium [2, 7]. The urothelial plaques or “asymmetric unit membrane” (AUM) (Fig. 1b) are constructed by uroplakins (UPs) (Fig. 1c), the markers of the advanced degree of the differentiation stage of umbrella cells [reviewed in [5, 8–11]. In mammals characterized by a thicker layer of the urothelium (*e.g*., humans), UPs are mostly present on the cell surface, in mammals with thinner urothelium (*e.g*. rodents), they can be also found in the deeper layers of the tissue [12]. UPs are highly tissue-specific, but in mice, some of their isoforms have been also identified in the mammary gland, pancreas, lung and heart [11]. Studies using transgenic models for UP and models with inactivated genes for these proteins provided important data regarding their function. Variation in their normal expression leads to a variety of urinary disorders such as vesicourethral, interstitial cystitis (IC), overactive bladder, hydronephrosis or renal dysfunction and renal failure [6, 11, 13, 14].

UPs, including immature form of UPII, are glycoproteins which are inextricably linked to their function. Disorders in normal glycosylation of UPs lead to abnormal epithelial adhesion, leakage of the urinary tract, increased tumor cell invasiveness and spread of *Escherichia coli* (*E. coli*) infection [15, 16]. However, little is known and understood how qualitative and quantitative changes in their sugar structures may influence pathological conditions of the urothelium. In depth knowledge of UPs glycosylation malfunctions might find applications in early diagnosis and targeted treatments of urinary tract pathologies. Presented review brings this fascinating but yet unexplored area in urothelium research.

## Uroplakins are the main structural components of urothelium

**Fig. 2:**
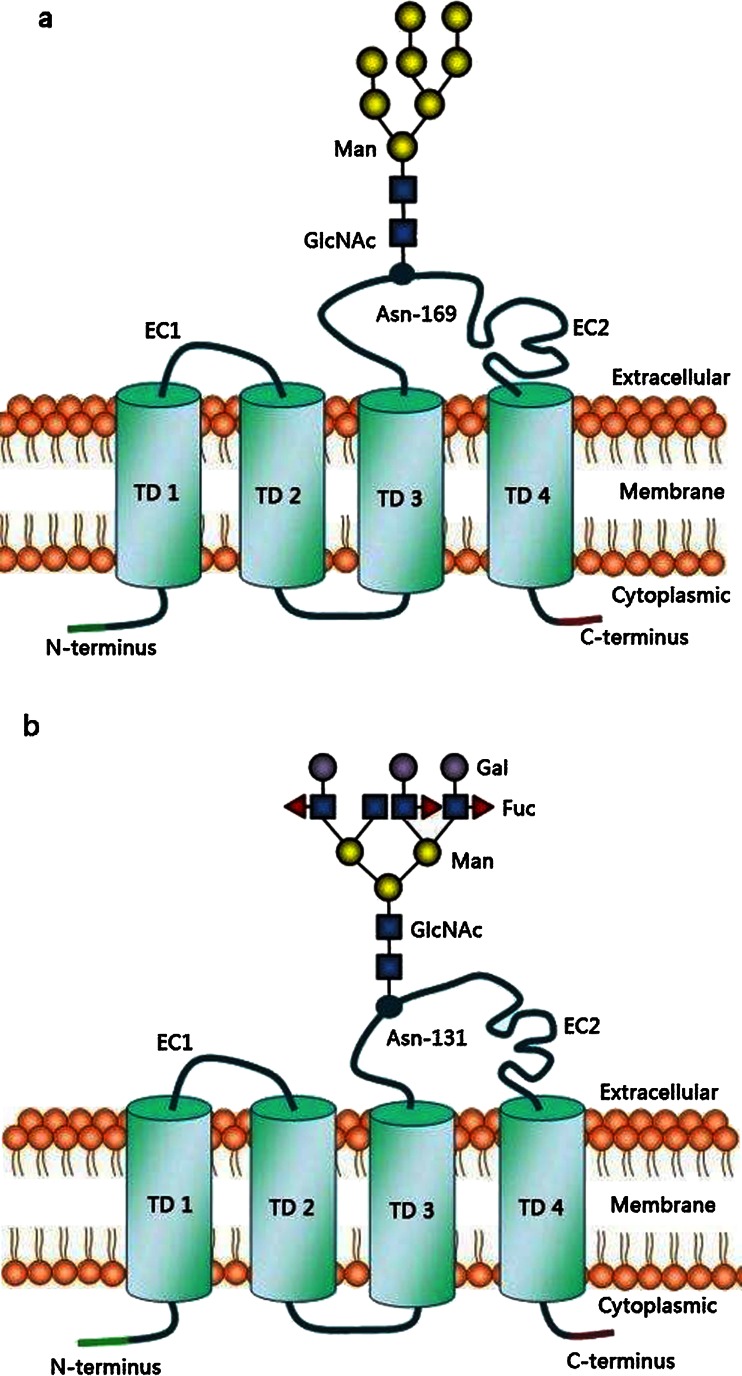
Sugar moieties on UPIa and Ib. UPIa and Ib possess four transmembrane domains (TD) (shown as cylinders) containing conserved aa residues. TD1 and TD2 are connected *via* a small extracellular loop (EC1), and TD3 and TD4 *via* a large extracellular loop (EC2). The main glycovariant of UPIa contain a high-mannose glycan linked to Asn-169 (**a**), whereas UPIb has a tetraantennary fucosylated complex glycan linked at Asn-131 (**b**). Abbreviations: *Asn* asparagine; *Gal* galactose; *GlcNAc* *N*-Acetylglucosamine; *Fuc* fucose; *Man* mannose

**Fig. 3:**
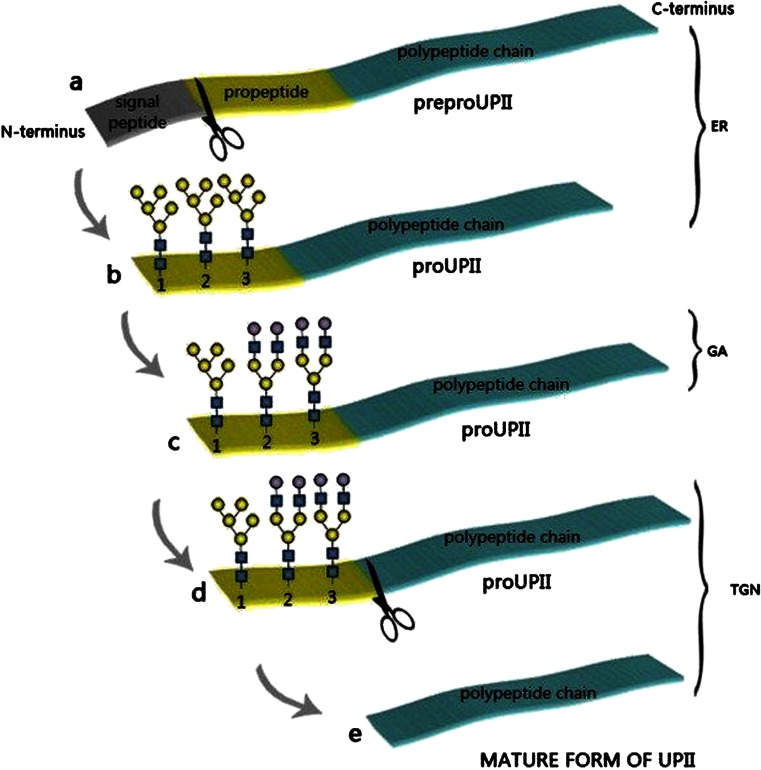
The dynamic glycosylation modifications during maturation of UPII. UPII is synthesized as a precursor prepropeptide (**a**). Cleavage of the signal peptide facilitates N-glycosylation of the propeptide by high-mannose glycans (**b**). The synthesis of preproUPII and signal peptide cut off take place in ER. In GA two of the three high-mannose N-glycans of the proUPII are transformed into complex glycans (**c**). Cleavage of the glycosylated propeptide occurs in TGN (**d**). The mature UPII lacks asparagine and thus sugar moieties (**e**). Abbreviations: *ER* endoplasmic reticulum; *GA* Golgi apparatus; *TGN* trans-Golgi network, 1-3- N-glycosylation sites. Drown based on [22]

AUM of all mammalian urinary bladder comprises four major UPs: UPIa and Ib, UPII, and III, which form a unique crystalline 2D array of 16-nm particles covering almost the entire polarized urothelial surface (Fig. 1) [17]. UPI a and Ib (27-kDa and 28-kDa, respectively) belong to the tetraspanin family. Their rod-shaped structures have four closely packed transmembrane helices (Fig. 2) spanning the membrane and extending into the extracellular loops, capped by a disulfide-stabilized head domain [18, 19]. In contrast, UPII and III (15-kDa and III 47-kDa, respectively) cross the membrane only once and their single transmembrane domain (Fig. 3) share a stretch of ~12 amino acid residues on the extracellular side [5, 8, 9, 20–22].

UPs are synthesized as monomers, however they undergo a dynamic and highly regulated assembly process, which begins in the endoplasmic reticulum (ER) where UPIa with UPII and UPIb with UPIII form specific cross-linked heterodimers [5, 8, 23, 24]. Formation of the UPIa/II and UPIb/IIIa heterodimers is a prerequisite for UPs to exit ER [24]. That stage of heterodimer formation seems to be functionally important as the ablation of UPsII or IIIa genes abolishes urothelial plaque formation and compromises their function [6, 13, 25]. However, Tu *et al*., [24] reported that UPIb can exit ER without a heterodimeric complex formation. In a post-Golgi compartment, UPs heterodimers form increasingly larger crystalline arrays. Two heterodimers bind to a heterotetramer through tight interactions of the transmembrane domains as well as the extracellular domains, so that the head domains of their tall partners can bridge each other at the top (Fig.1b). At turn, the interactions between the complexes and the tertiary interaction between the 16-nm particles contribute to the formation of UP tetraspanin structural/signaling networks. The aggregated UP complexes are sorted at the *trans*-Golgi network (TGN) into destined vesicles, which subsequently cover almost all mature fusiform vesicles in cytoplasm. These organelles migrate towards the apical cell surface where they fuse with the plasma membrane. By consequence, almost the entire apical urothelial surface is covered by 16-nm particles of urothelial plaques composed of UPs hexagonally packed into unique paracrystalline array [8, 18, 23, 26, 27]. The unique architecture of the hexagonal urothelial plaques with the distinctive cellular interconnections are critical for tightness, integrity and proper function of the urothelium [2, 5, 6, 11, 28, 29]. Little is known about the molecular events governing fusiform vesicles trafficking into the luminal surface and their incorporation into apical surface. Some molecules, such as a myelin-and-lymphocyte protein (MAL) expressed by T lymphocytes, myelin-forming cells, and polarized epithelial cells were reported to facilitate the incorporation of exocytic uroplakin-delivering vesicles into the apical membrane of urothelial umbrella cells [26, 27, 30–32].

UPs are tissue-specific molecules, but some of their isoforms have been also identified in other organs. For example in mice they have been found in the mammary gland, pancreas, lung and heart [11]. Adachi *et al*. [33] reported that UPIb is expressed in several non-urothelial epithelia, such as the corneal epithelium.

## N-glycans of UPs, O-glycoproteins and proteoglycans form urothelial glycocalyx

Urothelium is highly glycosylated tissue with luminal surface covered by glycocalyx composed of proteoglycans, N- and O- glycoproteins including secretory mucins. Quantitative analysis of the sialic acid, uronic acid, and hexosamine contents of delipidated rabbit bladder mucosa revealed a larger proportion of sialoglycoproteins as compared to glycosaminoglycans (GAG) [34].

The degree of glycosylation and microheterogeneity of the urothelium glycoproteins were reported to be associated and dependent on the origin of the species, the particular distribution in the multilayers of the urothelium, and the stage of the maturation and differentiation of urothelial cells. Using peanut agglutinin and *Vicia villosa* lectin as well as specific monoclonal antibodies, Vinter-Jensen and Ørntoft. [35] showed the presence of T and Tn antigens and mucin -type glycoproteins on murine and porcine urothelial tracts [35]. The expression of T and Tn glycotops on urothelium has been upregulated after stimulation of cell layers lining the urinary tract with epidermic growth factor (EGF) [35]. So far, certain mucins, such as MUC1 (epitectin) and proteoglycans, such as syndycan-1 have been isolated and characterized [36, 37]. As shown by Higuchi *et al.* [38] the rabbit mucins and glycoproteins of urothelium surface layers consisted of weakly sialylated and neutral oligosaccharides O-glycosidically linked to serine and threonine residues. In addition, the deeper layers of the rabbit urothelium (*e.i.*, lamina propria and muscle layer) are rich in GAGs, mainly hyaluronates and chondroitin sulfates, which presence on the surface of the urothelium are negligible [38]. By lectin-histochemistry, Desantis *et al*., [39] characterized sialylated O-linked glycans expressed on donkey (*Equus asinus*) urothelium. These glycans contained terminal or internal mannoses, and typical secretory moieties of sialylated O-glycoproteins (sialylated galactose and disaccharide composed of galactosamine-galactose). Expression of sialic acid on glycoproteins might indicate that the urothelium is not simply a inert barrier but can modulate the composition of soluble urinary proteins, which may play pathophysiological roles in the lower urinary tract [39]. Several urothelium-derived glycoproteins have been detected in urine of cows [1], rabbits [34, 36], pigs [40], rats [35] and people [37, 40, 41]. Using glycoproteomic technique Halim *et al*. [41] characterized 53 N-and O- glycoproteins out of 2,800 proteins present in human urine. They depicted 58 N- and 63 O- glycan profiles and urine complex type glycoproteins have been found to be sialylated and fucosylated bi- and multi- branched [41].

Closely related and conservative in their structure, transmembrane UPs types Ia/Ib (25-kDa/27-kDa), IIIa/IIIb (45-kDa/35-kDa) and immature form of UPII are N-glycoproteins [42, 43]. Their N- linked complex type oligosaccharides elicit microheterogenity. Thus, UPs can exist in several glycoforms, such as a high-mannose, bi- and/or more branched glycans, which could be further sialylated and/or fucosylated [42].

The function of urothelium glycoproteins can be related to those ascribed generally to the glycoproteins, and specific to the tissue. In the urothelium, their function is bound to the protection of membrane components from digestion by hydrolases, particularly present in the urine [40], participation in intercellular signaling, cell adhesion and recognition, selective permeability of molecules across the blood – urine barrier [1, 4, 10, 40, 44] and inhibition or promoting of bacterial colonization [42, 45]. There is some experimental evidence that unique architecture of urothelial plaques and function are strictly dependent of the UP glycosylation [5, 6, 15, 16, 21, 22].

## Glycosylation of UPIa and UPIb differs markedly

UPIa and Ib (25 and 27-kDa) are members of the tetraspanin family of glycoproteins with four transmembrane domains (TD 1–4) creating minor and larger loops, where the latter is stabilized by three disulfide bridges [19]. Both UPs are closely related, rich in charged amino acids approx. 40 % identical [4, 22, 46, 47]. Sugar parts of UPs Ia and Ib are located in the extracellular portion of their top surface (Fig. 2) exposed to the urothelium, in the hydrophilic larger loop connecting 3 and 4 TD domains [8].

In the 90s of the last century, based on data obtained by UPs N-glycosidase digestion it was assumed that both UPs: Ia and Ib on their extracellular surface domains carry high-mannose N-glycans, which protect protein part from digestion by proteases [8, 46, 48]. However, in 2001 Zhou *et al*. [14] reported that mouse UPs Ia and Ib, despite their identity in amino acid sequence, are glycosylated differently, and only UPIa harbors terminal mannose moieties, whereas the UPIb does not [14]. In 2006 Xie *et al*. [42] using more advanced techniques (permethylation of released glycans by in-gel glycosidase and protease digestion and mass spectrometric technique) confirmed that each of UPIs has only one potential N-glycosylation site. Mouse UPIa (25-kDa) possesses a glycan attached to Asn at 169 aa sequence position (Asn −169) (Fig. 2a) and UPIb (27-kDa) at 131 (Asn −131) (Fig. 2b). Murine and human UPIa are highly mannosylated with six to nine molecules of terminal mannose residues attached to *N*- acetylglucosamine core part (Man6GlcNAc2 and Man9GlcNAc2) (Fig. 2a) [42]. The predominant (43 %) murine glycovariant of UPIa contains eight terminal mannoses, whereas other glycoforms terminated by 7, 9 and 5 mannoses represent 28 %, 24 % and 5 %, respectively. In contrast, mouse UPIb is multi-antennary complex type oligosaccharide rich in fucoses (Fig. 2b). The fucosylated tetra-antennary N-glycoform is the main mouse UPIb glycovariant. A high-mannose variant of UPIb with 6 mannose residues and/or the hybrid type were found to be rare and constitute only 6 % to 15 % of UPIb [42].

The UPIa interact with the immature form of UPII (proUPII, *i.e*. containing a prosequence) whereas UPIb with UPIIIa forming UPIa/UPII and UPIb/UPIIIa heterodimers, respectively. That stage of dimer formation is important and facilitate their ER exit [22, 24, 49, 50]. Xie *et al*., [42] suggested that the drastically different carbohydrate processing of UPIa and UPIb proteins may reflect differences in their folding, masking and the interactions with associated proteins. The differences might be related to differences in accessibility of nascent proteins to glycosylation and/or alternatively to heterodimer formation. The initial step of N-glycosylation, a co-translational process is identical for both proteins *i.e*., the transfer of an N-glycan precursor, Glc3Man9GlcNAc2, from lipid-linked dolichol to a protein glycosylation site. The subsequent trimming of the precursor by alpha-mannosidases in ER and addition of other sugar moieties by glycosyltransferases in the Golgi complex [51, 52] vary significantly for UPIa and UPIb depending on peptide folding and accessibility of the glycosylation sites to glycosylating enzymes [42]. On the other hand, an alternative interpretation by Xie *et al*. [42] is that the prosequence portion of pro-UPII upon binding to its partner UPIa in ER can block UPIa’s single glycan from further trimming by the sugar-modifying enzymes.

It has been reported that mannosylated oligosaccharide part of UPIa can serve as the receptor for the FimH lectin adhesin of type 1-fimbriated *E. coli*, the bacteria that causes a great majority of urinary tract infections [11, 14, 42, 53]. This topic will be discussed in chapter below.

## Differentiation-dependent glycosylation of UPII

Based on sequence analysis of cDNA/gen and mRNA translation it has been established that UPII is primarily synthesized as a 19-kDa non-glycosylated prepro-UPII precursor (Fig. 3a). It comprises three parts of linked sequences: 1) a N-terminal 26–28 amino acids fragment (2-kDa), 2) a middle fragment located at 59 amino acids propeptide with three asparagines, the potential sites for N-glycosylation, and 3) a 100 amino acids polypeptide (15-kDa) corresponding to the mature native form of UPII [8, 20, 48]. The prepro-UPII precursor undergoes dynamic glycosylation modifications differentiation-dependent [5, 54], resulted probably from different participation of cellular glycosyltransferases [55]. In ER the signal sequence is cut-off from the 19-kDa prepro-UPII precursor. A propeptide is further glycosylated by addition of three attached high-mannose N-glycans. The attachment of sugar chains to pro-UPII causes an increase in the molecular weight of the immature form of UPII to 29-kDa (Fig. 3b). Further increase of molecular mass of proUPII to 32-kDa is associated with the transformation of two out of three high-mannose glycans into complex glycans (Fig. 3c) [5, 22, 24, 54]. Next, in TGN the glycosylated peptide is cut-off from pro-UPII (Fig. 3d) and mature form of UPII is created. The precursor of bovine pro-UPII which carries complex N-glycans does not contain exposed sites susceptible for degradation by exoproteases, but has four furino-like endoproteases. For efficient cleavage, the furin-cleavage site requires some arginines in signature sequence: Arg − 4-Xaa − 3-(Lys/Arg) − 2-Arg − 1 [56]. The released mature bovine UPII (15-kDa) does not possess the asparagines/s able to be N-glycosylated [20]. The mature UPII bound with a cell has a long extracellular domain of 71 amino acids, a transmembrane domain of 25 amino acids, and short intracellular domain [8, 20, 48]. It has been suggested that the furin-mediated cleavage of the glycosylated prosequence of UPII plays a role in regulating the assembly of UP to form AUM [22].

An analogous scheme for maturation of UPII was reported for UPII of nearly all mammals with the exception of mouse UPII where propeptide possesses one potential glycosylation site [48]. However, there is no data on whether mature form of mouse UPII expresses or not oligosaccharide parts.

The pro-UPII by itself cannot exit ER unless is stabilized by partner’s UPIa. [22, 24]. The glycosylation alternations are responsible for conformational changes in pro-UPII that allow heterodimer formation. According to Hu *et al*., [22], the high-mannosyl-glycans on pro-UPII appear only in ER and later, the mannosylated glycans are further processed in Golgi network. In consequence two of them become the antennary complex type oligosaccharides. Moreover, Hu *et al*., [57] indicated that the prosequence of UPII requires a proper disulfide linkage in order to maintain hairpin-like proUPII conformation which facilitate binding to UPIa and the formation of the proUPII/UPIa complex. It has been reported that the induced conformational changes allow UPIa/proUPII complex to exit ER and facilitate further proUPII maturation [5, 22, 24, 54]. Hu *et al*. [22] hypothesized that a special, complex glycan-forming glycosyltransferase in superficial umbrella cells might be involved in producing AUM-associated 32-kDa pro-UPII, which in turn can promote the binding of two partner’s heterodimers (UPIa-UPII and UPIb-UPIII) to form a heterotetramer.

The exclusive expression of all high-mannose glycans on pro-UPII, and lack of the antennary complex N-glycans, can exclude a heterotetramer formation and finally can cause a lack of AUM on the surface of umbrella cells [22]. Moreover, the high-mannose N-glycans of pro-UPII participate in preventing the crystalline plaques assembly in undifferentiated intermediate cells of urothelium [22]. Interestingly, the differentiation-dependent glycosylation of pro-UPII does not occur in cultured bovine urothelial cells, and the synthesized UPs do not assemble into crystals in cultured cells [54].

## UPIII comprises a large N-linked oligosaccharide moiety

**Fig. 4:**
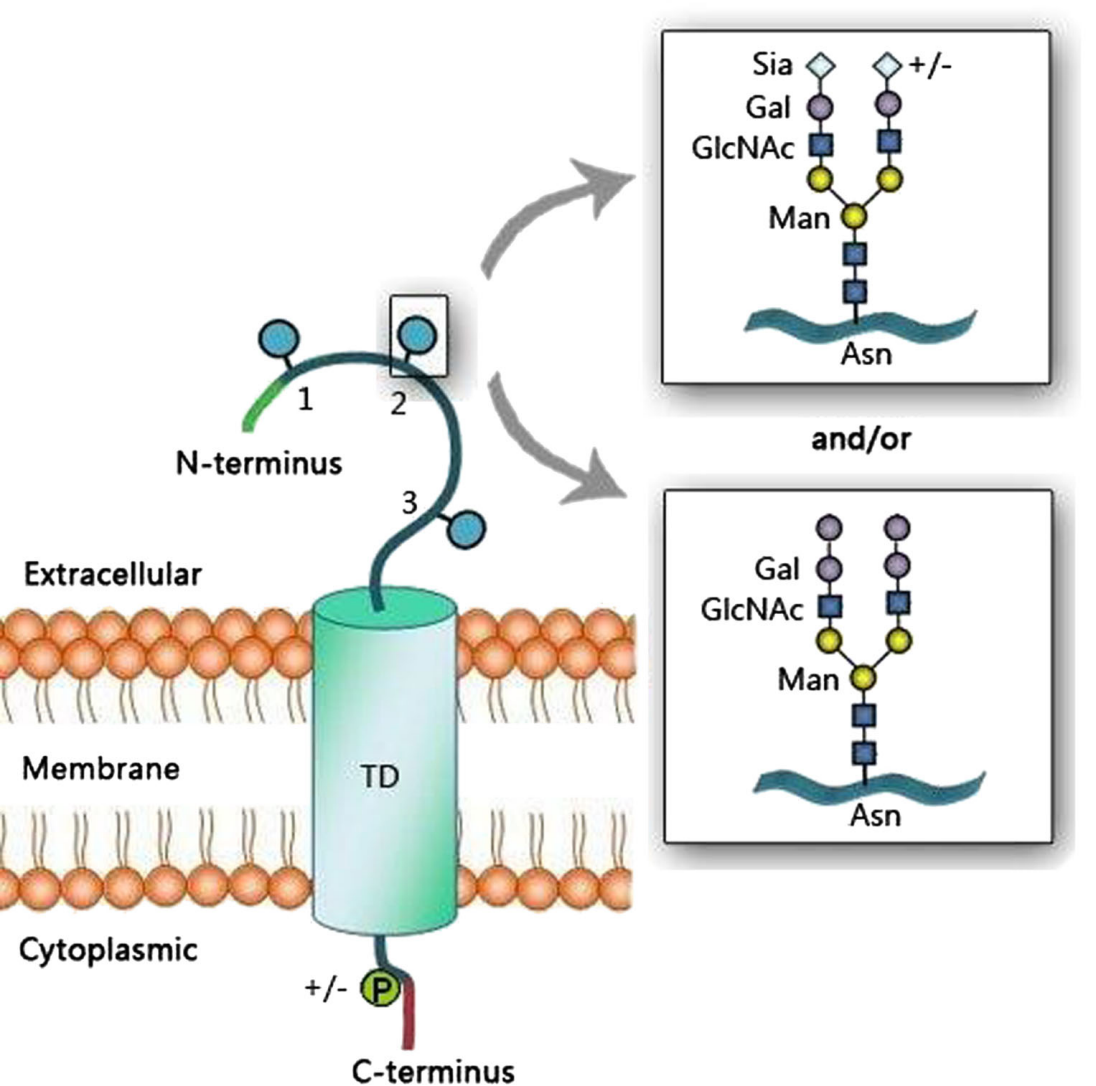
Hypothetical glycosylation of UPIIIa. UPIIIa is highly glycosylated and possess complex N-glycans (blue circles). Bovine UPIII N-glycans may contain Sia linked to Gal *via* α(2-3)- or α(2-6)-glycosidic bonds. Cellular signaling occurs *via* phosphorylation of the UPIIIa cytoplasmic tail. Drown based on [9] and [58]

UPIII (UPIII 47 to 49-kDa) is a single, typical integral transmembrane glycoprotein with three potential N-glycosylation sites (Fig. 4) and cysteine residues on N-terminal extracellular domain (189 amino acids). UPIII carbohydrates significantly contribute to the surface glycocalyx [9]. Charged residues of a shorter, C-terminal cytoplasmic part of UPIII (52 amino acids) have multiple potential phosphorylation sites. Deglycosylation and cDNA sequencing revealed that UPIII contains a large N-linked oligosaccharide moiety of up to 20-kDa attached to a 28-kDa-core protein.

The composition of sugar part of UPIII is species dependent. The polypeptide part of bovine UPIII consists of 287 amino acids (28.9-kDa), and its large sugar moiety constitute more than 40 % of the total mass of the whole molecule [9, 48]. This sugar moiety is larger than glycans expressed by other uroplakins. The deglycosylation of bovine glycoprotein by specific N-glycosidase F (PNGase F) and O- glycosidase digestions indicates that UPIII contains N-linked glycan/s and lacks O-linked glycan/s [9]. Based on cDNA sequence analyses, Wu and Sun [9] hypothesized that UPIII sequence contains four potential N-glycosylation sites. They pointed out three N-glycosylation sites at Asn34, Asn74, and Asn170 [9]. They also suggested that the glycosylation at Asn34 is questionable because of a proline [9]. So far, there is no experimental data showing which of predicted glycosylation sites are truly glycosylated. Malagolini *et al*. [58] showed that N-oligosaccharides of bovine UPIII can be terminated by sialic acid α(2-3)- and α(2-6)-linked to Gal(1-4)-GlcNAc-sequence, however some other glycovariants may contain the terminal Galα(1-3)-Gal epitope formed by galactose residue α(1-3)-linked to the Galβ(1-4)-GlcNAc unit of antennary N-glycan (Fig. 4). The occurrence of the Galα(1-3)-Gal epitope on N-glycans in UPIII is species specific and such epitope is absent on UPIII and other human N-glycoproteins. Enzyme α1,3-galactosyltransferase is not expressed in humans, apes and Old-World monkeys where the gene is inactivated through frame shift and nonsense mutations [58].

Hu *et al*., [6] reported that the UPIII ablation leads to the overexpression, defective glycosylation, and abnormal targeting of UPIb. Interestingly, also UPII ablation leads to a similar up-regulation of the UPIb mRNA level and its hypoglycosylation suggesting that UPIb changes represent a general response to a perturbed uroplakin assembly [13]. The UPIII-depleted urothelium features small plaques, becomes leaky, and has enlarged uretheral orifices resulting in the back flow of urine, hydronephrosis, and altered renal function indicators. Deng *et al*., [50] reported that in the cases of UPIII ablation, UPI is able to form a dimer with a novel 35-kDa urothelial plaque associated glycoprotein (p35) that is closely related to UPIII [50]. The synthesis of UPIII-related glycoprotein is most likely up-regulated as a compensatory mechanism in the case of UPIII absence. The UPIII-related glycoprotein and UPIII have a similar overall type 1 transmembrane topology. Their amino acid sequences are 34 % identical, share an extracellular juxtamembrane stretch of 19 amino acids and the both UPIII isoforms form a heterodimer with uroplakin Ib, but not with any other UPs. Deng *et al*., [50] devided p35 UP into uroplakin IIIa (47–49-kDa) and IIIb (35-kDa). In spite of fairly low levels of UPIIIb (10 % of UPIIIa), its interaction with UPIb in ER is believed to be an important early step in urothelial plaque assembly [50]. It has been found that UPIIIb is expressed in urothelial tumors of the urinary bladder in cows that had suffered from chronic enzootic hematuria for several years [59]. Given the fact that the protein parts of the UPIIIa and IIIb have similar molecular masses (approximately 30-kDa), both isoforms of UPIII can differ in the content of glycosylated and/or phosphorylated residues attached [50].

Recent work by DeSalle’a *et al*. [60] concerning the divergent evolution of uroplakin, indicated the existence of additional mammalian isoform - UPIIIc. According to the authors, the UPIIIc amino acid sequence is 37 % identical to a protein part of UPIIIb and like two other UPIII isoforms can form heterodimers with UPIb. In contrast to other UPIIIs, UPIIIc isoform does not have a ~20 aa region between the transmembrane domain and the N-terminus of the protein [60]. So far, nothing is known about glycan moiety structure of UPIII isoforms other than UPIIIa.

## Mannose residues on UPIa are pivotal for urinary tract *E.coli* infection

Urinary tract infection involving *E. coli* constitutes 90 % of all infections of this system and the second most common in the human population infectious disease with high treatment costs [53, 61, 62]. Infection is initiated by attaching bacteria to the normally sterile uroepithelium *via* a lectin- type 1-fimbriae (FimH) adhesins of *E.coli* to urothelial surface receptor UPIa, rich in mannose residues [14, 63, 64]. The reaction is specific for *E. coli* expressing adhesins H -type, and not *E. coli* with other types of adhesins or bacteria lacking fimbria [42, 65]. Effective interaction of FimH with UPIa may also cause infections of the upper part of the urinary tract including kidneys since expression of UPIa has been also found in the ureters and renal pelvis [66–68]. In these locations a significant role in bacterial adhesion is attributed to fimbriae P expressed by certain strains of *E. coli* causing pyelonephritis [69]. Malagolini *et al*. [58] reported that initiation of infection in cows is also possible *via E. coli* strains expressing S-type fimbriae able to recognize α(2-3)-sialylated glycans expressed by bovine α2,3-sialylated UPIII glycovariant. After successful invasion, uropathogenic bacteria replicate, invade neighbouring cells, and mature into dense, biofilm-like inclusions. Within the bladder epithelium bacteria can survive for months and are responsible for treatment- challenging recurrent urinary tract infections [53, 63, 64].

Group of Wu *et al*. [45] for the first time showed that type 1-fimbriated *E. coli* binds to bovine urothelial plaque proteins *via* UPIa. Later, in view of significant differences in the glycosylation between UPIa (high mannosylated glycan) and UPIb (almost exclusively complex-type glycan), the former one has been recognized as responsible for *E. coli* infections [14, 42, 70, 71]. The multimannosylated glycoforms of UPIb present in minute quantities are buried deeply in the structural pocket and are not available for bacterial adhesins [42].

The urothelial receptor on UPIa for bacterial adhesin FimH was localized by Min *et al*. [71] on the six inner domains of the 16 nm urothelial plaque particle. FimH adhesin has a mannose-binding pocket that is capable at pH 4–9 of specific reaction with glycoproteins with exposed mannose residues [14, 70, 71]. FimH binding with UPIa occurs with a moderate strength (Kd ~100 nM), but a multiple binding sites present on the bacteria and multiple array of polymerized UPs receptors, makes the attachment relatively strong [14]. Wellens *et al*., [63] reported that FimH interacts with Manα(1-3)Manβ(1-4)GlcNAcβ(1-4)GlcNAc in an extended binding site. The interactions along the α(1-3) glycosidic bond and the first β(1-4) linkage to the chitobiose unit are conserved with those of FimH with buty α-D-mannose. The strong stacking of the central mannose with the aromatic ring of Tyr48 is congruent with the high affinity found for synthetic inhibitors in which mannose is substituted by an aromatic group [63].

The adhesion of uropathogenic *E. coli* to highly mannosylated UPIa is only an initial step of subsequent events in the pathogenesis of urinary tract infections [64, 72, 73]. Studies of Wang *et al*., [64] indicated that the interaction of lectin-mediated adhesion with the urothelial UPIa can further induce cellular signaling by phosphorylation of the UPIIIa cytoplasmic tail and leads to cytoskeletal rearrangements and further bacterial invasion. They showed by cryo-electron microscopy that FimH binding to the extracellular domain of UPIa induces global conformational changes and movements in all UPs transmembrane helices. Downstream signaling events can be initiated by lateral translocation of the UP cytoplasmic tails [64]. Thumbikat *et al*. [73] further discovered that equally important to efficient insult by *E. coli* and bacterial adhesion is the UPIIIa cytoplasmic tail phosphorylation on a specific threonine residue by casein kinase II, followed by an elevation of intracellular calcium. Moreover, the same group [72] utilizing *in vitro* models of urothelial differentiation, demonstrated that *E. coli* mediated cell-death is entirely dependent on the proper differentiation of UPIII and directly correlated with its enhanced maturation. Thus, during the urinary tract infections where superficial urothelial cells are damaged and less differentiated cells are exposed, urothelial apoptosis is reduced [72]. Further, Klumpp *et al*. [74] using a murine model of urinary tract infection showed that urothelial apoptosis is a key event in the pathogenesis mediated by *E.coli* and requires both caspase-dependent direct induction of the extrinsic pathway and caspase 8-mediated Bid cleavage -dependent indirect intrinsic pathway [74].

Against excessive colonization by microorganisms, AUM is protected by glycocalyx formed of glycosylated UPs [5, 10, 75]. Experimental work by Geerlings *et al*. [76] showed that people with diabetes are more prone to urinary tract infections compared to healthy people. It is therefore suggested that disorders in glycosylation of UPs found for example in diabetes, may have a significant role in the development of urinary tract infections, especially in their recurrent and persistent form [15, 77]. Like diabetes, bacterial adhesion facilitates transportation of immatured mannosylated glycoproteins to the surface [16]. It has been proven that the expression profile of sugar residues expressed by UPs is more important for the epithelial adhesion of microbes than the variations of an individual determinant of bacterial adhesin [78].

Attempts to block interactions between bacterial adhesins and sugars exposed by UPs are of great importance for unraveling the extremely important clinical issue related to bacterial infections of urothelium. It should be noted that the physiological concentrations of naturally occurring mannosylated glycoprotein present in urine (Tamm - Horsfall protein) are able to inhibit adhesion of bacteria and serve as a first line of defense against infection [79, 80]. In recent years, many new revolutionary antibacterial agents that effectively eliminate urinary tract infection have been designed. A particularly promising class of drugs include remedies that inhibit bacterial adhesion to urothelial cells *i.e*. FimH antagonists for example in the form of vaccines, which, by their mechanism of action minimize bacterial drug resistance [81–84]. Design of FimH antagonists, precisely glyco-agonists are based on the imitation of natural sugar epitope structures [63, 83, 85]. Recently, Schwardt *et al*. [51] attempted to create new FimH antagonists on the structure of triazoles with mannose residues. In other work, *in vitro* and *in vivo* studies showed that a protein D present on the surface of the mucous membranes of various organs for example in lungs, has the ability to bind directly to UPIa and block bacterial adhesion to the urothelium [86]. Preventing and treating of urinary tract infections is one of the most important priority of modern medicine because of the universality and continuous increase in the incidence of this medical condition.

## Impairment of the mucosal glycoconjugates in IC

An impairment of the mucosal glycoconjugates could be an important factor in the development of bladder disorders such as interstitial cystitis (IC). The disease is characterized by increased permeability of urothelial membrane, influx of urine into underlining tissues, lower back pain and frequent, urgent and painful voidings [29]. Epidemiological studies indicate that this disease is much more common than recognized clinically. A number of theories have been created to explain the etiology of IC and the data cited by various authors are sometimes contradictory [87, 88]. One of the most important and most popular theories proposed by Parsons is the concept of increased permeability of the urothelium to harmful substances in the urine that are linked to quantitative and qualitative changes in the layer of GAGs [89]. Parsons *et al*. showed reduced secretion of GAGs and one of their metabolite - uronic acid in urine of IC patients as compared to control group [90]. The observations made using a scanning electron microscope confirmed the reduction in the GAG layer and leakage of urothelial membrane. The concept of increased permeability of urothelial cells was further supported by findings of Fowler *et al*., which revealed in patients the presence of Tamm-Horsfall protein deposits [91]. Later, Neal *et al.* established the presence of antibodies against Tamm-Horsfall protein in the patient serum [92]. The majority of patients demonstrated qualitative changes of proteoglycans [88]. These findings again underline a crucial role for the proper composition of glycovariants and precise glycosylation of the urothelium in the function and physiological condition of the urothelial tract.

## Glycosylation changes in urothelial cancer

Cancer derived from the urothelium is the sixth most common type of cancer in the world. About 90–95 % of cases occur in the bladder, the remaining involve the ureter and renal collecting system (about 5–10 %). In cancer, the cancer-associated carbohydrate structures are reported to play key roles in cancer progression by altering the cell-cell and cell-environment interactions. As a consequence of neoplastic transformation the cell membrane glycoconjugates undergo characteristic changes [93, 94]. UPs create attractive urothelium tumor markers due to the high conservation of their structure and selective tissue specificity. However, it seems somewhat paradoxical that their expression does not exactly correlate with the degree of differentiation of cancer and they might be found even in metastatic tumors despite the fact that they are final differentiation products of normal urothelial cells [49, 95]. On the other hand, exploration of the differentiation–dependent glycosylation of uroplakins might shed the light on this conundrum.

So far little is known how glycosylation changes correlate with dedifferentiation of cancer cells, the stage and progression of the disease [16]. Pode *et al*. [96] showed that expression of Lewis X glycoantygen detected on the umbrella cells is associated with bladder cancer with specificity of 86.4 %. The tumor-associated carbohydrate, an antigen sialyl-Tn (STn) and its major biosynthetic enzyme, the sialyltransferase (sT6), highly expressed by several human carcinomas and preneoplastic lesions [93] have been reported to be expressed by tumors of the bladder [97, 98]. Ferreira *et al*. [97] showed that 75 % of the high-grade bladder tumours, presenting elevated proliferation rates and high risk of recurrence/progression, expressed STn glycotope. The antigen STn has not been found in the normal urothelium, but was mainly expressed in non-proliferative areas of the tumour invading the basal and muscle layers as well in tumour-adjacent mucosa suggesting a tumor field effect. Furthermore, the data of Lima *et al*. [98] strongly suggested that Bacillus Calmette–Guerin (BCG) immunotherapy is efficient against STn - and s6T-positive tumours. Further authors proposed that expressions of that glycoantigen and its related enzyme may be used as independent predictive markers of BCG treatment response and in the identification of patients who could benefit from this immunotherapy. Recently, Zupančič *et al*. demonstrated in rodents a modified expression of species- dependent sugar moieties during carcinogenesis [16]. Using lectin-immunohistochemical analysis they showed *in vivo* and *ex vivo* that rodent neoplastic urothelium displayed higher reactivity with *Jacalin* (lectin from *Artocarpus integrifolia*) as compared to normal tissue. Unlike in mouse carcinogenesis model, in rat model the decreased binding to another natural *Amaranthus caudatus* lectin was noted. Moreover, in normal urothelium, terminally differentiated umbrella cells expressed all four UPs, which were present in the large urothelial plaques covering mature fusiform vesicles and the apical plasma membrane. In contrast, the preneoplastic urothelium contained poorly differentiated cells with microvilli and small, round vesicles that were uroplakin-negative and no urothelial plaques were observed in these cells [16].

UPII and particularly UPIII are considered as highly tissue-specific and moderately sensitive markers for primary and metastatic urothelial cancers, metastatic cancer of unknown primary origin, and markers that differentiate primary carcinoma of the urothelium from other cancers of the genitourinary system [5, 10, 59, 99–110]. UPIII is also considered as valuable and sensitive marker appearing in urine in association with bladder cancer [108]. However, none of the research team analyzed the alterations in the expression/s of glycotope/s on UPs in relation to cancer of the bladder and/or urinary tract. Future studies and insight into UP glycoforms as differential diagnostic and early indicators of urotelial cancer should be encouraged.

Besides the diagnostic usage for glycovariants of UPs, studies of their glycoconjugates carry important applications in the future for development of targeted drugs through lectins. A specific structure of oligosaccharide moieties of the urothelium glycoconjugates has been tested for improved intravesical drug delivery in cancer therapy [111, 112]. The idea of glycotargets approach of Neutsch *et al*. [111, 112] is based on a molecular efficient cytoinvasion of uropathogenic bacteria, mediated *via* a mannose-directed FimH adhesin, and malignancy-dependent differences in bladder cell glycosylation. Their carrier-based delivery concept, combines biorecognitive targeting using plant lectin wheat germ agglutinin (WGA) immobilized on poly(lactide-co-glycolide) microparticles for steady cytoadhesion. Several DNA-selective chemotherapeutics with established track record in uro-oncology for physicochemical compatibility with the polymeric carrier formulation have been tested and the preliminary data are promising. The results of this work show potential in glycotargeted deliveries in the intravesical setting and offer new perspectives for the application of lectin-based drugs in the urinary tract malfunctions [112]. However, progress on drug delivery systems tailored to the penetration-hostile urothelial barrier lags behind the advancements in comparable fields.

## Conclusions and future prospects

Particularly fascinating and newly discovered aspect of uroplakin biology and thus the biology and physiology of the urothelium are uroplakin glycovariants. It seems that an in-depth knowledge of the molecular pathogenesis of glycosylation, formation of microheterogenic glycoforms, products of their metabolism and degradation may set new directions for future research. Some of glycotopes terminated by sialic acid and/or fucose as tumor markers could potentially be used to detect tumors in asymptomatic patients, especially at high risk or monitoring of recurrence after treatment of the primary tumor.

Besides of diagnostic usage, the glycoconjugates can carry important applications in lectin-mediated drug delivery [16]. Blood- urine barrier which forms the urothelium is one of the least permeable membranes of the body and is also a challenge to the penetration of drugs. Studies by Kreft *et al*. showed that the WGA lectin binds to and is endocytosed by umbrella cells at hinge regions, a potential entry for biopharmaceuticals [113, 114]. The use of lectins recognizing the sugar residues at this particular region can be very effective and valuable in construction of targeted therapies and the treatment of infectious diseases, cancer and IC of the urinary system. Such selective delivery of the active ingredient (drug) directly to urothelium allows targeted treatment without creation of aberrant cell damage to healthy tissues.

There is no doubt that glycosylation of UPs is closely related to UPs function and its malfunction is a reflection of the ongoing pathological process. Due to the fact that changes in the glycosylation of UPs are primary to the underlying changes in relation to the overall expression of the UP, their early detection may help to capture lesions in their early stages. Since urine analysis has a number of advantages, including the availability of large quantities of the material and noninvasive sampling, testing glycobiomarkers in urine is highly desirable [115]. In order to evaluate and validate the clinical utility of UPs glycoforms, their variable molecular native forms and/or possibly degraded, mannosylated versus sialylated and fucosylated glycovariants, further studies on the large scale and the involvement of multidisciplinary research teams are needed.

## Abbreviations

Asn: asparagine
Gal: galactose
GlcNAc: *N*-Acetylglucosamine
Man: mannose
Fuc: fucose
P: phosphate
Sia: sialic acid
TD: transmembrane domain
1-3: N-glycosylation sites
